# Associations Between Developmental Stability, Canalization, and Phenotypic Plasticity in Response to Heterogeneous Experience

**DOI:** 10.1002/ece3.70436

**Published:** 2024-10-22

**Authors:** Shu Wang, Ragan M. Callaway

**Affiliations:** ^1^ College of Forestry, Forest Ecology Research Center Guizhou University Guiyang China; ^2^ Division of Biological Sciences, Institute of Ecosystems The University of Montana Missoula Montana USA

**Keywords:** fluctuating asymmetry, inter‐individual variation, intra‐individual variation, leaf size, temporal heterogeneity, total mass, water availability

## Abstract

The processes of developmental stability, canalization, and phenotypic plasticity have ecological and evolutionary significance, and been studied extensively, but mostly separately and thus the relationships between them are not straightforward. Our objective was to better integrate these processes in the context of temporally heterogeneous environments. We did this by investigating the effects of early experience with temporal heterogeneity in water availability on associations between developmental stability, canalization, and phenotypic plasticity. We subjected eight plant species to a first round of alternating inundation and drought vs. constantly moderate water treatments (heterogeneous experience) and a second round of water conditions (to test plasticity). We measured fluctuating asymmetry (FA) in leaf size, intra‐ and inter‐individual variation (CV_intra_ and CV_inter_), and plasticity (PI) in traits and analyzed correlations between these variables across all species. Results showed little correlations between FA, CV_intra_ and PI, several positive correlations between FA and CV_inter_ in more stressful conditions, especially in as well as positive correlations between CV_inter_ and PI initially and negative correlations between them later. These suggested the complexity of these relationships, which can depend on whether plasticity occurs. Greater inter‐individual variation will more likely cooperate with plasticity before or during plastic response, whereas higher canalization may reflect phenotypic convergence. Both higher FA and CV_intra_ can reflect faster growth, while CV_intra_ may also reflect plant growth stage, and the two mechanisms should cooperate in response to environmental challenges. The complexity of these relationships suggests plants deal with environmental variation in elaborate and integrative ways which can be affected by many factors.

## Graphical abstract

Decreased canalization may promote plastic responses in traits before or during the induction of plasticity, whereas canalization may reflect phenotypic convergence after plastic responses.

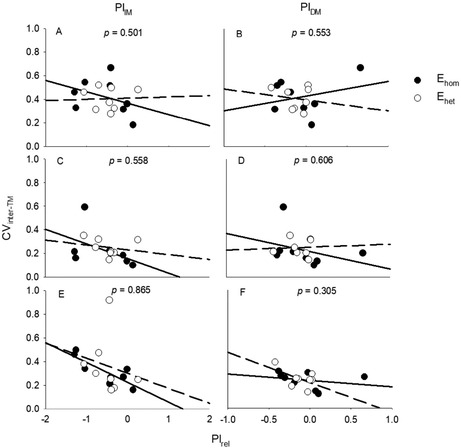

## 1 Introduction

Environmentally induced phenotypic variation is an important source of variation for selection, and thus of evolutionary and ecological significance (Bradshaw 1965; Debat and David 2001; Miner et al. 2005; Via et al. 1995), despite once being regarded as randomly generated or a nuisance for understanding genetic rules (Darwin 1859; Falconer 1952). The three most frequently studied processes that contribute to phenotypic variation are phenotypic plasticity, developmental stability, and canalization (Debat and David 2001) (see Table 1). Phenotypic plasticity, the ability of a genotype to produce different phenotypes in different environmental conditions (Bradshaw 1965), is central to the emergent field of ecological evolutionary developmental biology (“eco‐evo‐devo”; Pfennig 2016). Developmental stability (Bradshaw 1965) and canalization (Waddington 1942) are thought to counteract phenotypic plasticity. Developmental stability (or instability) is the ability of an individual to buffer its development against genetic or environmental disturbances and produce a predictable phenotype (Auffray, Debat, and Alibert 1999; Clarke 1998; Zakharov 1992). This is usually evaluated through measurements of fluctuating asymmetry (random deviation from perfect symmetry of bilateral traits) or intra‐individual variability of traits (Møller and Swaddle 1997; Woods et al. 1999). Canalization, including environmental canalization and genetic canalization, is the ability to buffer development against genetic or environmental perturbations by reducing phenotypic variation (Stearns, Kaiscr, and Kawecki 1995; Wagner, Booth, and Bagheri‐Chaichian 1997; Wilkins 1997), and is often measured by inter‐individual variation (coefficient of variation in traits) (Woods et al. 1999). Although both developmental stability and canalization can lead to phenotypic consistency (Zakharov 1992), canalization differs, in that it is to some extent modifiable (Waddington 1961) or developmentally flexible (Thoday 1953) due to autoregulatory mechanisms (Schmalhausen 1949). This suggests that developmental systems can be both phenotypically plastic and stable at the same time. Robustness or developmental buffering are often used as synonyms for canalization (De Visser et al. 2003; Félix and Wagner 2008) or developmental stability (Lempe et al. 2013), while developmental sensitivity is similar to phenotypic plasticity (Masel and Siegal 2009; Mestek Boukhibar and Barkoulas 2016; Wagner 2007).

Because of their interrelationships, research on developmental stability, canalization, and phenotypic plasticity has increased over the last two decades (Debat and David 2001; Hammelman et al. 2020; Saito, Tsuboi, and Takahashi 2024; Tucić et al. 2018). It is reasonable to believe the ability of an organism to change and to maintain stability should function simultaneously to guide development and regulate interaction with the environment (Bradshaw 1965; Debat and David 2001). However, studies of their such simultaneous function have reported inconsistent results (Debat and David 2001; Flatt 2005). For example, the underlying mechanisms for developmental stability and canalization have been reported as independent (Debat et al. 2000) but also as related (Debat, Debelle, and Dworkin 2009; Lazić et al. 2016). Plasticity and developmental stability can correspond (Willmore, Klingenberg, and Hallgrimsson 2005; Woods et al. 1999) or not (Debat et al. 2000; Milton et al. 2003), or even be negatively related (Hammelman et al. 2020; Saito, Tsuboi, and Takahashi 2024). Thus studies shed light on possible connections between developmental stability, and canalization as well as between them and plasticity, but direct evidence is lacking (Tonsor, Elnaccash, and Scheiner 2013; Tucić et al. 2018; Wang and Zhou 2022a, 2022b), leaving an intriguing knowledge gap in our understanding of how plants respond to temporal variation in the environment (Pfennig 2016). Investigations of intra‐individual variation in general remain scarce, and we know little about the relationship of intra‐individual variation to fluctuating asymmetry and thus developmental stability. We know even less about the connection of intra‐individual variation to canalization and plasticity. Contradicting results suggest that relationships among developmental stability, canalization, and plasticity are very complex and depend on specific traits, environmental contexts, and growth stages (Mustafić and Freund 2013; Takahashi 2019; Woods et al. 1999).

Temporal heterogeneity in resource availability is ubiquitous in nature, but most studies have focused on spatial heterogeneity despite plasticity often being very high in temporally dynamic habitats (De Meester 1996; Gianoli and González‐Teuber 2005; Lázaro‐Nogal et al. 2015). Moreover, previous exposure to temporally heterogeneous hydrological conditions can improve later growth potential of species by modifying their plasticity (Wang and Callaway 2022). It is unknown whether temporally heterogeneous environments affect developmental stability or canalization, or the connections of these processes to plasticity. If plasticity can be modified by heterogeneous environments, we might be able to identify associations among the processes by tracing changes in plasticity, or by comparing relationships among the processes before and after plastic responses. Plants provide excellent opportunities for such studies because they are sessile and thus may rely on these processes to cope with environmental changes (Bradshaw 1965; Sultan 2000). However, up to now, studies of the relationships between developmental stability, canalization, and plasticity have focused mostly on animals (Tonsor, Elnaccash, and Scheiner 2013; Tucić et al. 2018), and analogous studies of plants are rare.

We conducted an experiment with eight plant species, four native and four exotic to North America, to investigate how experience with temporally heterogeneous water supply influences developmental stability, canalization, and plasticity later in development (defined as plastic response at later stage) to water conditions. We also measured correlations among these processes before and after plastic responses to answer the following questions: (1) Does early exposure to temporally heterogeneous water availability affect the plasticity, fluctuating asymmetry, intra‐variability (developmental stability) and inter‐individual variability (canalization) of traits? (2) Are there correlations between these processes, and do they differ before and after plastic responses? And (3) Does early heterogeneous experience alter these correlations?

**TABLE 1:** Information for the concepts of developmental stability, variability, canalization and phenotypic plasticity, and associated traits used in this study.

Concept	Definition	Evaluation	Abbrev.	Calculation	Trait
Phenotypic plasticity	The shift in phenotype due to changes in environments (Schlichting 1986)	The difference (with direction) in a given trait due to environmental effects (Valladares, Sanchez‐Gomez, and Zavala 2006)	PI_rel_	PI_rel_ = (*X − Y*)/(*X* + *Y*) where *X* was the adjusted mean trait value of plants in an environment, and *Y* was the adjusted mean value in another environment (Valladares, Sanchez‐Gomez, and Zavala 2006)	Shoot mass (SM) Root mass (RM) Total mass (TM) Root to shoot ratio (R/S)
		The difference (with no direction) in a given trait due to environmental effects (Valladares, Sanchez‐Gomez, and Zavala 2006)	PI_abs_	PI_abs_ = |(*X − Y*)/(*X* + *Y*)| where *X* was the adjusted mean trait value of plants in an environment, and *Y* was the adjusted mean value in another environment (Valladares, Sanchez‐Gomez, and Zavala 2006)	
Developmental stability	The tendency of traits to resist the effect of developmental errors (Palmer and Strobeck 1986)	Fluctuating asymmetry—random deviation from perfect bilateral symmetry (Møller and Swaddle 1997)	FA	FA_1_ = ∑|*R − L*|/*n* FA_2_ = ∑[(*R − L*)/*S*]/*n* FA_10_ = 0.798 × √*s* ^*2*^ where *s* ^2^ = (MS_sj_–MS_m_)/*M*, *R* and *L* were the width of right and left sides of a leaf, *n* was the total number of leaves, and *S* (leaf size) was calculated by (*R* + *L*)/*2* (Palmer and Strobeck 1986, 2003)	Leaf size (LS)
		Intra‐individual coefficient of variation (Woods et al. 1999)	CV_intra_	The standard deviation divided by mean value of the trait within an individual	Leaf size (LS)
Canalization	The ability of a genotype to produce consistent phenotypes regardless of environmental and genetic variabilities (Waddington 1942)	Inter‐individual coefficient of variation (Woods et al. 1999)	CV_inter_	The standard deviation divided by mean value of the trait for all individuals within a population	Leaf size (LS); Shoot mass (SM) Root mass (RM) Total mass (TM) Root to shoot ratio (R/S)

## 2 Materials and Methods

### 2.1 Species

We used four exotic invasive species—*Leucanthemum vulgare* Lam. (oxeye daisy), *Centaurea stoebe* L. ssp. *micranthos* (Gugler) Hayek (spotted knapweed; née *C. stoebe* L.), *Leonurus cardiaca* L. (common motherwort), and *Potentilla recta* L. (sulfur cinquefoil). We compared these to four native species—*Heterotheca villosa* (Pursh) Shinners (hairy false golden aster), *Gaillardia aristata* Pursh (blanket flower), *Agastache urticifolia* (Benth.) Kuntze (nettleleaf giant hyssop) and *Potentilla arguta* Pursh (tall cinquefoil) (Table 2). Three pairs of exotics and natives shared families and one pair were congeners. All seeds were collected from natural grasslands in western Montana. The distributions of these species can overlap, but we selected groups of target species that generally occur in habitats that share positions on a soil moisture gradient. *C. stoebe* and *H. villosa* are broadly distributed but expand into more xeric habitats, *L. vulgare*, *P. recta*, *G. aristata*, and *P. arguta* overlap in distribution with the first two, but are less abundant in more mesic habitats, and *L. cardiaca* and *A. urticifolia* are generally found in wetter habitats.

**TABLE 2:** Summary of the attributes of the eight species studied and abbreviations for their names.

Latin name	Abbrev.	English name	Family	Invasiveness	Moisture range of habitats
Centaurea stoebe L. ssp. micranthos (Gugler) Hayek	C	Spotted knapweed	Compositae	Invasive	Mesic~xeric
Leucanthemum vulgare Lam.	Lv	Oxeye daisy	Compositae	Invasive	**Mesic~xeric**
Potentilla recta L.	Pr	Sulfur cinquefoil	Rosaceae	Invasive	**Mesic~xeric**
Leonurus cardiaca L.	Lc	Common motherwort	Lamiaceae	Invasive	**Hydric~mesic**
Heterotheca villosa (Pursh) Shinners	H	Hairy false golden aster	Compositae	Native	Mesic~xeric
Gaillardia aristata Pursh	G	Common gaillardia	Compositae	Native	**Mesic~xeric**
Potentilla arguta Pursh	Pa	Tall cinquefoil	Rosaceae	Native	**Mesic~xeric**
Agastache urticifolia (Benth.) Kuntze	A	Nettleleaf giant hyssop	Lamiaceae	Native	**Hydric~mesic**

*Note:* The coefficients in bold font indicate significance at < 0.10 level.

### 2.2 Experimental Design

The experiment was conducted in a greenhouse at the University of Montana, Missoula, Montana. Greenhouse temperatures were maintained between 15°C and 30°C, corresponding roughly to natural summer temperatures in the region. Natural light was supplemented by metal halide bulbs and maximum total photosynthetically positive radiation on clear days reached ~1200 μmol m^−2^ s^−1^. Seeds of all species were sown in plastic trays (54.2 × 27.3 cm in width and 6.5 cm in height) in December 2010. Two weeks after seedling emergence, individual seedlings were transplanted into pots (7 × 7 cm in width and 20.6 cm in height) filled with a 1:1 mixture of top garden soil and sterile silica sand. Forty days after transplanting, before the first round of treatments, the longest leaf of each plant was measured as an estimate of the initial size of each individual. A split plot design was implemented with the first round of treatments as a main factor and the second round of treatments and species as sub‐factors. There were two “early experience” treatments: alternative inundation‐drought as an early heterogeneous treatment (E_het_), and consistent moderate watering as an early homogeneous treatment (E_hom_, control). The design of 2 cycles of environmental treatments allows comparing the relationships between variables before (in the first cycle) and after (in the second cycle) plastic responses are induced, thus will be helpful for finding the reasons for variation in these relationships. For each species, a subgroup of 20 individual plants from these treatments was harvested after 90 days for measurements. The remaining plants from each of the two early treatment groups were divided into three subgroups, each of which was later exposed to either inundation, moderate watering, or drought treatments (Figure S1). For each treatment combination in the second round, eight species with 10 individuals for each species were used (20 individuals per species were measured from each treatment in the early treatment). In sum, with one individual per pot, and 10 replicates × 8 species × 2 early treatments × 3 late treatments + 20 replicates × 8 species × 2 early treatments = 800 pots in total.

### 2.3 Experimental Treatments

Six identical tanks were used to create alternative inundation‐drought conditions and moderate water conditions, with three tanks assigned to each of the two treatments. Tanks were 161 × 91.3 cm in width, 8.5 cm in height, lined with heavy plastic, and fitted with drains to regulate maximum water depth. The E_het_ treatment was implemented by first subjecting plants to a round of inundation for 2 weeks, then another round of drought for another 2 weeks, followed by 2 weeks of inundation again, for a total of three periods of 2‐week inundation mixed with 2‐week drought experiences. For the E_hom_ treatment, we watered each pot daily to capacity, and soil remained moist throughout the experiment. The first round of treatments lasted for 90 days before subsets of plants in each treatment were either divided and placed into three different conditions for the second round or harvested and measured to evaluate early responses (Figure S2). For the inundation treatment in the first round of E_het_ treatment and the second round, the water level was maintained at 7 cm depth above the bottom of the tank, approximately 10 cm below the surface of the soil in the pots, and pots were also watered to saturation every day. There was no standing water in the moderate and drought treatment tanks, but in the moderate treatment (the first round of E_hom_ treatment and the second round), pots were watered to saturation every other day. Pots in the drought treatment (in the first round of E_het_ treatment and the second round) were watered to saturation once or twice per week. This varied as we tried to stress plants without killing them. In this second round of treatments, six tanks were used to create inundation, drought, and moderate water conditions, with two tanks assigned to each treatment. The duration of the second round of treatments was 60 days, and greenhouse and hydrological conditions were as similar as possible to the first round. Plants receiving both rounds of treatments were harvested and measured after 150 days, separated into roots and shoots, dried at 60°C for 2 days, and weighed.

### 2.4 Data Collection

We calculated mortality rates for all treatment combinations. Across all treatments and species, 720 individuals survived to the end of the experiment and were used for further analyses. Traits of total mass, shoot mass and root mass and root to shoot ratio were used to assess the performance of species in the first and second rounds of treatments.

Developmental stability was evaluated by the degree of deviation of leaf width from perfect asymmetry (i.e., fluctuating asymmetry; see below). Canalization was evaluated as the coefficient of variation (CV, the standard deviation divided by mean value of the trait) among individuals (CV_inter_) in leaf size and mass traits (Debat and David 2001). CV in leaf size among different leaves on single individuals (CV_intra_) was used to evaluate developmental variability or intra‐individual variability (Woods et al. 1999). Canalization includes environmental canalization and genetic canalization. Since we used individual seeds from a small local population, the measures of canalization in traits mainly reflect environmental canalization. The processes of developmental stability, or variability, and canalization are thought to reflect individual‐level and population‐level stabilities, respectively (Debat and David 2001).

Both the level and degree of plasticity (relative and absolute plasticity) in mass traits were calculated using the Simplified Relative Distance Plasticity Index (Valladares, Sanchez‐Gomez, and Zavala 2006), which was abbreviated as PI. We calculated relative plasticity (PI_rel_) in traits with index (1) and absolute plasticity (PI_abs_, the degree of plasticity) with index (2), with formulas as follows:

(1) $${\mathrm{PI}}_{\mathrm{rel}}=\left(Y_{2}–Y_{1}\right)/Y_{1}$$

(2) $${\mathrm{PI}}_{\mathrm{abs}}=|\left(Y_{2}–Y_{1}\right)/Y_{1}|$$

where *Y*_2_ represented the adjusted mean trait values in late inundation or drought for each species after early heterogeneous or homogeneous treatment, and *Y*_1_ represented adjusted mean trait values in moderate water conditions after the same early treatment. PI_IM_ and PI_DM_ were used to represent plasticity in response to inundation and drought vs. moderate conditions respectively. Adjusted mean values for all traits were produced from one‐way ANCOVA on original mean values, with late water conditions as effect and initial size as a covariate.

### 2.5 Estimation of Developmental Stability

For each individual, we measured all the leaves on the main stem, immediately after plants were removed from pots, in a random sequence for all samples of all treatments. To calculate leaf fluctuating asymmetry (FA), the width of right and left halves (from the midrib to the margin) for each leaf was measured twice successively and immediately after each other, at the widest point of the leaf (perpendicular to the midrib) with a digital caliper (Wilsey et al. 1998). The leaf size (LS) was calculated as the average width of right and left sides (Palmer and Strobeck 1986; Wilsey et al. 1998). We compared various conventional indexes (FA_1_–FA_8_ and FA_10_) in calculating FA, to identify the indexes with the highest explanatory powers for our study design (Table S1). Different indexes showed similar trends in response to water conditions, thus we adopted FA_1_, FA_2_ (with and without effects of leaf size respectively), and FA_10_ (the only index with measurement error variance partitioned out of the total between‐sides variance) in analyses, with the formula (Palmer 1994; Palmer and Strobeck 2003) as:

(3) $${\mathrm{FA}}_{1}=\sum |R–L|/n$$

(4) $${\mathrm{FA}}_{2}=\sum \left[\left(R–L\right)/\mathrm{LS}\right]/n$$

(5) $${\mathrm{FA}}_{10}=0.798\times \sqrt{\left({\mathrm{MS}}_{\mathrm{sj}}-{\mathrm{MS}}_{m}\right)/M}$$

where *R* and *L* were the width of right and left sides of a lamina, *n* was the total number of laminas, LS (lamina size) = (*R* + *L*)/*2*, MS_sj_ was the mean squares of side × individual interaction, MS_m_ was the mean squares of measurement error, and *M* was the number of replicate measurements per side, from a side × individual ANOVA on untransformed replicate measurements of *R* and *L*.

The |*R* – *L*| was regressed on LS for all the leaves of individuals per species in each treatment to determine the size‐dependence of leaf asymmetry, and most cases of leaf asymmetry were size‐dependent (Table S2). We measured skew (*γ*_1_) and kurtosis (*γ*_2_) to evaluate whether the leaf asymmetry deviated from normality. To detect the presence of antisymmetry, kurtosis (*γ*_2_) was tested with a *t*‐test of the null hypothesis H_0_: *γ*_2_ = 0, where a significant negative *γ*_2_ indicates possible antisymmetry (Cowart and Graham 1999; Palmer 1994). To test the presence of directional asymmetry, we used two methods: (1) testing (*R* – *L*) against 0 with one‐sample *t*‐test (the hypothesis H_0_: *γ*_1_ = 0); and (2) testing whether the difference between sides (mean squares for side effect [MS_s_]) is greater than nondirectional asymmetry (mean squares for side × individual interaction [MS_si_]) with factorial ANOVA (Palmer 1994; Wilsey et al. 1998). Only one set of samples (G in E_hom_) showed leptokurtosis, indicating antisymmetry, and three sets of samples showed right‐dominated directional asymmetry and one showed left‐dominant directional asymmetry. More than half of the (11) sets of samples also showed greater mean difference between sides (MS_s_) than between‐sides variation (MS_si_; Table S3), indicating directional asymmetry. We also evaluated whether between‐sides variation is significantly larger than the measurement error (MS_m_) in factorial ANOVA (Palmer 1994). The MS_m_ values for all cases were much lower than MS_si_ values.

### 2.6 Statistical Analysis

All variables for traits were used in statistics, and the original data was log‐transformed before any analysis to minimize variance heterogeneity. All analyses were conducted with SAS statistical software (SAS Institute 9.0 Incorporation 2002). Three‐way ANCOVA was performed for overall effects of early experience, habitat type, nativity, and their interactions on all variables, with initial size (IS) as a covariate. Then one‐way ANCOVAs were used for the effects of early experience and nativity on all variables within each of the other treatments combined and across all the other treatments, with initial size as a covariate. Multiple comparisons used the Least Significant Difference (LSD) method in General Linear Model (GLM) program for mean values of LS, FA, CV, and PI indexes. We also used CV equality R package (Marwick and Krishnamoorthy 2019) to compare CV values between 1st‐round or 2nd‐round treatments for each species. For each treatment and across all treatments in the three experiments, correlations among LS, FA, CV, and PI indexes across all species were analyzed with PROC CORR, producing Pearson Correlation Coefficients (PCC) for correlations of LS with FA, CV and PI indexes, and Partial Pearson Correlation Coefficients (PPCC) for correlations among FA, CV, and PI indexes with LS or CV_inter_ in IS as a covariate.

## 3 Results

### 3.1 Direct Effects of Early Heterogeneous Experience on Leaf FA and Trait CV

After the 1st round of treatments, pooled across all data, early treatments had significant effects on leaf size (Table S4), and exotic and native species differed in leaf size, leaf fluctuating asymmetry (FA_1_, FA_2_) and inter‐individual coefficient of variation (CV_inter_) in shoot mass (CV_inter‐SM_; Table 3; Table S5).

**TABLE 3:** Pearson partial correlation coefficients (PPCC) for correlations among 1st‐round variables including leaf size (LS), fluctuating asymmetry (FA_10_), CV_intra_ and CV_inter_ of leaf size (CV_intra‐LS_, CV_inter‐LS_) and CV_inter_ of total mass (CV_inter‐TM_) across all species in early temporally homogeneous (E_hom_) and heterogeneous (E_het_) treatments, with effects of initial size in control.

Variable	E_hom_				E_het_			
	LS	CV_intra‐LS_	CV_inter‐LS_	CV _inter‐TM_	LS	CV_intra‐LS_	CV_inter‐LS_	CV _inter‐TM_
LS		0.201	−0.409	0.204		−0.083	−0.253	0.314
FA_10_	0.928***	0.186	−0.163	0.450	0.951***	−0.150	0.756**	0.498
CV_intra‐LS_			−0.140	−0.549			−0.246	−0.708*
CV_inter‐LS_				0.495				0.554

*Note:* Abbreviations for all traits are in Table 1. *p < 0.10, **p < 0.05, ***p < 0.01. The coefficients in bold font indicate significance at < 0.10 level.

**FIGURE 1:**
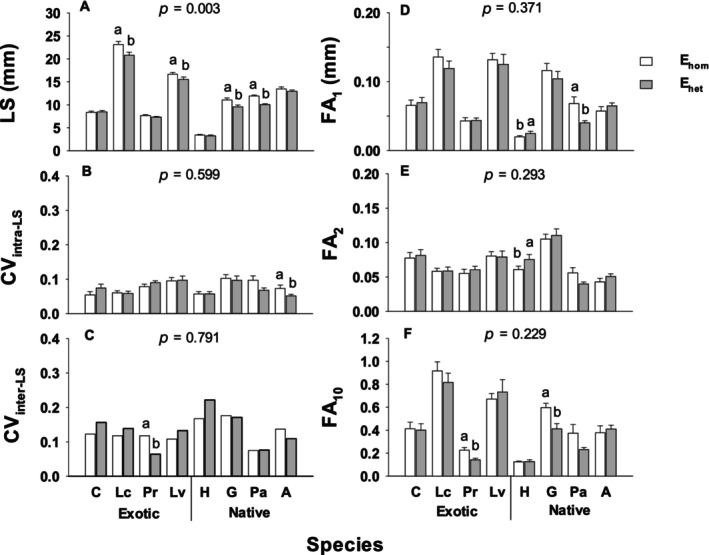
Mean values (±SE) of leaf size (LS, A) and its intra‐ and inter‐individual coefficient variation (CV_intra_ and CV_inter_, B, C) and fluctuating asymmetry (FA_1_, FA_2_, FA_10_, D–F) of leaf size for eight species in the 1st round of heterogeneous (E_het_) and homogeneous (E_hom_) treatments. Different letters indicate significant differences between E_het_ and E_hom_ treatments for each species (*p* < 0.05). The abbreviations for all species are in Table 2.

**FIGURE 2:**
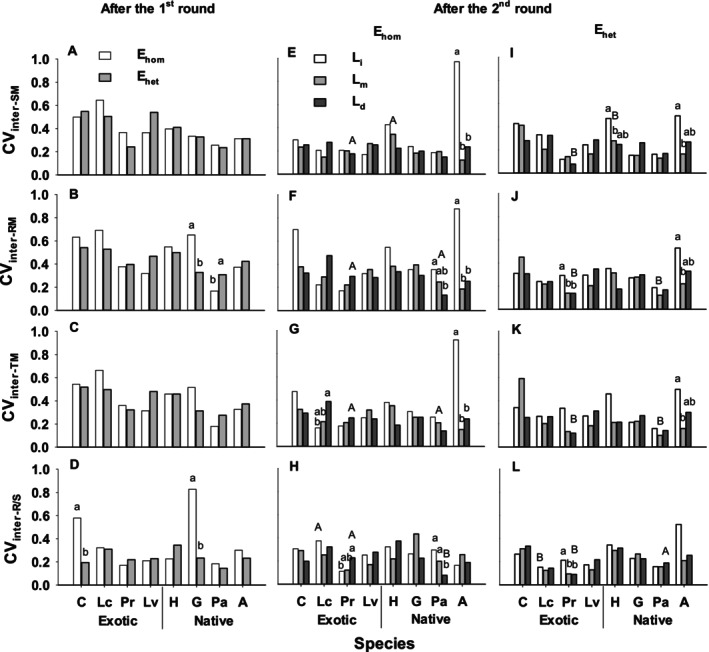
Inter‐individual coefficient of variation (CV_inter_) in shoot mass (CV_inter‐SM_, A, E, I), root mass (CV_inter‐RM_, B, F, J), total mass (CV_inter‐TM_, C, G, K) and root to shoot ratio (CV_inter‐R/S_, D, H, L) in the 1st‐round treatments (A–D), and in the 2nd‐round inundation (L_i_), moderate (L_m_), and drought (L_d_) conditions (E–L) for eight species (grouped by exotics vs. natives) with early homogeneous (E_hom_, E–H) and heterogeneous (E_het_, I–L) experience. The abbreviations for all species are in Table 2.

For individual species, compared to the early homogeneous treatment (E_hom_, control), the early heterogeneous treatment (E_het_) decreased leaf size of *Leonurus cardiaca*, *Leucanthemum vulgare*, *Potentilla arguta*, and *Gaillardia aristata* (*p* < 0.010; Figure 1). E_het_ also increased FA_1_ and FA_2_ of *Heterotheca villosa* (*p* < 0.050), decreased FA_1_ of *P. arguta* (*p* = 0.026) and FA_10_ of *P. recta* and *G. aristata* (*p* < 0.010), and intra‐individual coefficient of variation (CV_intra_) in leaf size (CV_intra‐LS_) for *Agastache urticifolia* (*p* = 0.050) and CV_inter‐LS_ for *P. recta* (*p* = 0.011). Compared to controls, E_het_ decreased mean values of root mass, total mass, and root: shoot ratio of *P. arguta* and *G. aristata* (*p* < 0.050; Figure S3), and decreased CV_inter‐RM_ and CV_inter‐RS_ of *G. aristata* (*p* = 0.024 and *p* < 0.001) and CV_inter‐RS_ of *Centaurea stoebe* (*p* = 0.040), but increased CV_inter‐RM_ of *P. arguta* (*p* = 0.016; Figure 2).

Comparing species in groups, exotic species had higher mean leaf size, FA_1_ and FA_10_ than native species after either E_hom_ or E_het_ (*p* < 0.050), while natives had higher CV_intra‐LS_ than exotics after E_hom_ (*p* = 0.015; Figure S4), but lower CV_inter‐RM_ and CV_inter‐TM_ than exotics after E_het_ (*p* = 0.020 and *p* = 0.053; Figure S5).

### 3.2 Later Effects of Early Heterogeneous Experience on Trait PI and CV

After the 2nd round of treatments, early experience had significant effects on CV_inter‐RM_ (Table S5), but no effect on plasticity in any traits (Table S6). However, for individual species, compared to E_hom_, E_het_ increased mean plasticity in response to late inundation vs. moderate conditions (PI_IM_) in mass traits for *L. vulgare* and *A. urticifolia* (*p* < 0.05), increased the plasticity in response to late drought vs. moderate conditions (PI_DM_) in these traits for *L. vulgare* and *G. aristata* (*p* < 0.01), but decreased the mean PI_DM_ of *L. cardiaca* and *P. recta* (*p* < 0.01; Figure S6).

Compared to E_hom_, E_het_ decreased CV_inter‐SM_, increased CV_inter‐TM_ for native species in late moderate conditions (Figure 2). For individual species, compared to E_hom_, E_het_ increased the performance of mass traits in different late conditions for several species (*p* < 0.050; Figure S3), but decreased CV_inter_ in mass traits of several species (*p* < 0.050; Figure 2). Compared to E_hom_, E_het_ decreased CV_inter_ in all mass traits in late drought for *P. recta* (*p* < 0.050) and CV_inter‐RM_ and CV_inter‐TM_ for *P. arguta* (*p* < 0.050) and CV_inter‐SM_ of *H. villosa* in late moderate conditions (*p* < 0.039; Figure 2). *Agastache urticifolia* had higher CV_inter_ for mass traits in late inundation vs. other conditions after E_hom_ (*p* < 0.050), but had higher CV_inter_ in these traits in late inundation vs. moderate conditions after E_het_ (*p* < 0.050). *Leonurus cardiaca* had higher CV_inter‐TM_ in late drought vs. inundation after E_hom_ (*p* = 0.028), whereas *H. villosa* had higher CV_inter‐SM_ in late inundation vs. moderate conditions after E_het_ (*p* = 0.017). In terms of grouped species, compared to E_hom_, E_het_ decreased CV_inter‐SM_ in late moderate conditions for native species (*p* = 0.028; Figure S5).

### 3.3 Correlations Between Different Variables

In the 1st‐round treatments, FA_10_ correlated positively with leaf size after either E_hom_ or E_het_, whereas CV_intra‐LS_ positively correlated with FA_10_ and negatively correlated with CV_inter‐TM_ after E_het_ (Table 3). In the 2nd‐round treatments, CV_inter‐TM_ in late drought (CV_LD_) had positive correlations with leaf size, FA_10_, and CV_inter‐TM_ in the first round after E_hom_. CV_inter‐TM_ in later drought had positive correlations with FA_10_ after either early experience (Table 4). CV_inter‐TM_ in late moderate water conditions also positively correlated with the first‐round CV_intra‐LS_ after E_het_.

**TABLE 4:** Pearson partial correlation coefficients (PPCC) for correlations of 2nd‐round CV_inter_ in total mass (CV_inter‐TM_) with 1st‐round variables including mean values of leaf size (LS), fluctuating asymmetry (FA_10_), CV_intra_ and CV_inter_ of leaf size (CV_intra‐LS_ and CV_inter‐LS_), and CV_inter_ of total mass (CV_inter‐TM_) in late inundation (L_i_), moderate (L_m_), and drought (L_d_) treatments across all species with previous experience of temporally homogeneous (E_hom_) or heterogeneous (E_het_) conditions, with effects of initial size or CV_inter_ in initial size (CV_inter‐IS_) in control.

Variable	E_hom_			E_het_		
	L_i_	L_m_	L_d_	L_i_	L_m_	L_d_
LS	−0.191	−0.446	0.689*	−0.181	0.063	0.633
FA_10_	−0.371	−0.217	0.722*	−0.280	0.216	0.777**
CV_intra‐LS_	−0.061	−0.052	−0.026	−0.303	0.718*	0.171
CV_inter‐LS_	0.228	0.265	0.071	−0.209	−0.185	0.357
CV_inter‐TM_	−0.483	0.090	0.720*	−0.178	0.223	0.474

*Note:* Abbreviations for all traits are in Table 1. *p < 0.10, **p < 0.05. The coefficients in bold font indicate significance at < 0.10 level.

**FIGURE 3:**
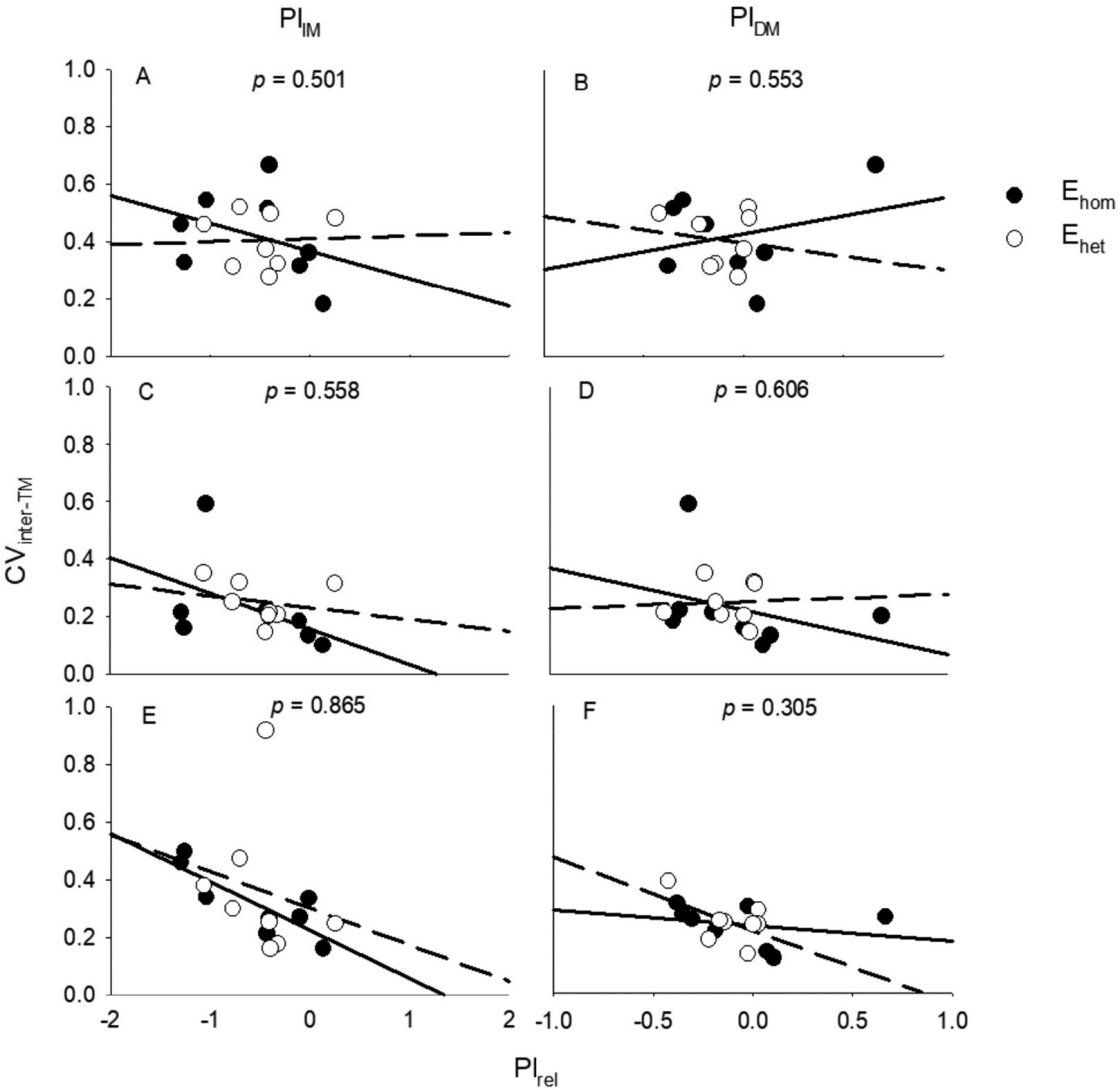
Regressive relationships of relative plasticity (PI_rel_) in total mass in response to inundation (PI_IM_, left) or drought (PI_DM_, right) vs. moderate conditions with inter‐individual coefficient of variation (CV_inter_) in total mass (CV_inter‐TM_) in the 1st‐round treatments (A, B), and in the 2nd‐round moderate (C, D), inundation (E), and drought (F) conditions across all species with early homogeneous (E_hom_, solid circle with solid line) and heterogeneous (E_het_, empty circle with dotted line) experience. The *p*‐values indicate the significance of difference in slopes between two early experiences.

CV_inter‐TM_ in the first round had positive correlation with PI_IM_, CV_inter‐TM_ in late inundation had negative correlations with PI_IM_ after E_hom_ or E_het_, and CV_inter‐TM_ in late drought had positive correlations with PI_DM_ after E_hom_ (Table 5; Figure 3).

**TABLE 5:** Pearson partial correlation coefficients (PPCC) for correlations of 2nd‐round relative plasticity (PI_rel_) in total mass in response to inundation (IM) or drought (DM) vs. moderate conditions with 1st‐round variables including mean values of leaf size (LS), fluctuating asymmetry (FA_10_), CV_intra_ and CV_inter_ of leaf size (CV_intra‐LS_ and CV_inter‐LS_), and CV_inter_ of total mass (CV_inter‐TM_) in late inundation (L_i_), moderate (L_m_), and drought (L_d_) treatments across all species with previous experience of temporally homogeneous (E_hom_) or heterogeneous (E_het_) conditions.

Round	Variable	E_hom_		E_het_	
		IM PI_rel_	DM PI_rel_	IM PI_rel_	DM PI_rel_
1st round	LS	0.263	0.226	0.365	−0.641
	FA_10_	0.199	0.038	0.334	−0.585
	CV_intra‐LS_	0.494	−0.403	0.443	0.167
	CV_inter‐LS_	−0.665	−0.494	−0.284	0.109
	CV_inter‐TM_	−0.025	0.019	0.840**	−0.189
2nd round	CV_inter‐TM_ in L_i_	−0.754**	—	−0.832**	—
	CV_inter‐TM_ in L_m_	0.584	0.311	0.150	−0.265
	CV_inter‐TM_ in L_d_	—	−0.828**	—	−0.422

*Note:* Abbreviations for all traits are in Table 1. **p < 0.05. The coefficients in bold font indicate significance at < 0.10 level.

## 4 Discussion

### 4.1 Responses of Different Variables

#### 4.1.1 Phenotypic Plasticity

Scheiner and Holt (2012) predicted that temporal environmental variation occurring after development and before selection favors the evolution of plasticity, using an individual‐based computer simulation model. Within‐generation heterogeneity can result in cue unreliability (Scheiner 2013), and variation and uncertainty affect whether or not plasticity is favored (Scheiner and Holt 2012). Our results showed that temporally heterogeneous water conditions will increase the degree of plastic responses of plants to inundation, but decrease plasticity in response to drought. In any case, the effects of temporally heterogeneous experiences have been shown to be positive in terms of plant performance (Wang and Callaway 2022). Temporally heterogeneous environments likely increase biochemical and physiological traits of plants that improve later performance and growth, whereas temporally homogeneous experiences such as shading tend to increase biomass and morphological traits initially, but at the cost of reduced growth potential later in development (Wang et al. 2023). Temporal heterogeneity in environmental conditions may trigger mechanisms that cope with stress and prepare plants for later growth and adaptation.

#### 4.1.2 Fluctuating Asymmetry

Compared to early constant moderate‐water experience (control), experience with alternating inundation and drought conditions (temporally heterogeneous experience) decreased the leaf size of three species, suggesting such an experience was stressful, in terms of current performance (Wang and Callaway 2022). In comparison, responses of fluctuating asymmetry (FA) in leaf size were mixed among species. FA decreased in response to heterogeneous experience for two species, increased for one species, and was not changes for the other species. Higher FA is thought to reflect developmental instability, often indicating stress (Gonzalez, Lotto, and Hallgrímsson 2014; Hagen et al. 2008; Møller 1998). However, others report that FA may be an unreliable indicator of environmental stress (Abeli et al. 2016; Palmer 1994; Palmer and Strobeck 2003), and the relationship between FA and environmental conditions is not always clear among traits, species, or functional groups (Bonduriansky 2009). Other studies also report that higher FA or developmental instability can suggest that organisms or modules are growing rapidly in favorable environments (Martel, Lempa, and Haukioja 1999; Morris et al. 2012), such as high nutrient or water availability (Milligan, Krebs, and Mal 2008), less polluted soil (Velickovic and Perisic 2006), or less intense competition (Wang and Zhou 2022b). However, shading can increase cotyledon FA, perhaps due to its facilitative effects as it induces larger leaf area (Wang and Zhou 2022a). Therefore, our results suggested that higher FA is a response to less stress. Under more favorable conditions (or moderate stress, such as shading), faster‐growing individuals should be more able to increase performance in traits, and thus have higher leaf FA (Wang and Zhou 2022b). In other words, developmental instability can be beneficial in some circumstances.

#### 4.1.3 Intra‐Individual Variation

Intra‐individual variation (CV_intra_) is one kind of intraspecific variability, which also includes intra‐population or inter‐individual variation and sub‐individual variation, all of which can have important ecological implications for individuals, populations, and communities (Herrera 2017; Wang and Zhou 2022b). Intra‐individual variability can affect how plants deal with environmental heterogeneity (Wang and Zhou 2022c), and thereby species distributions, and population stability and persistence (Wang and Zhou 2022b). For individual species, we found several cases of decreases in CV_intra_ in traits in response to temporal heterogeneity in the second round of treatments, consistent with similar decreases in response to increased density (Wang and Zhou 2022b). These results suggest that CV_intra_ resembles FA, indicating developmental stability or variability. And temporal heterogeneous water conditions and increased density were stressful to some extent for four of the eight species. Pélabon et al. (2021) found that within‐individual variation in seed size of *Dalechampia scandens* did not increase under stress, but weakly increased with unpredictable precipitation. It may be because the environmental unpredictability was not stressful for the species, but to some extent induced positive responses in plants.

#### 4.1.4 Inter‐Individual Variation

Temporal heterogeneous experience did not affect or decreased the inter‐individual variation (CV_inter_) in leaf size and mass traits, immediately or at the later stage for all species; and CV_inter_ decreased with drier conditions for four of the eight species. This suggests that environmental stress can decrease CV_inter_, and increase variation among individuals, and that this can be an advantage. Inter‐individual variation can be either advantageous or disadvantageous, depending on specific circumstances, for example, the property or strength of environmental stress (Wang and Zhou 2021, 2022b). More intense or unpredictable stress will more likely enhance inter‐individual variability in populations due to the increase in the number of smaller plants (Valladares et al. 2002), whereas more moderate or predictable stress may reduce or have no effect on inter‐individual variability as a result of decreased or canalized growth rate (Wang and Zhou 2022b). The treatment of alternating inundation and drought, or temporal heterogeneity in water supply, may be less stressful than constant inundation or drought, and thus decrease or not affect inter‐individual variation (Wang and Callaway 2022). For example, Wang and Zhou (2022b) reported increased CV_inter_ in traits with increased density for in more fertile soil at later stages, due to stronger stressful effects of increased density. The intensity of a given stress often differs for different species; thus, temporally heterogeneous experience does not reduce inter‐individual variation for all species (Wang and Zhou 2022a).

### 4.2 Correlations Between Different Variables

#### 4.2.1 Correlation Between Developmental Stability and Plasticity

Mechanisms for developmental stability and plasticity correlate with each other (Willmore, Klingenberg, and Hallgrimsson 2005; Woods et al. 1999; Tonsor, Elnaccash, and Scheiner 2013; Tucić et al. 2018). We did not find significant correlations between FA and PI or CV_intra_ and PI, but positive correlation between FA and PI has been found for plants across a range of densities and growth stages (Wang and Zhou 2022b). Evidence for stronger correlations between these mechanisms should be expected when stronger positive or negative responses are induced (Wang and Zhou 2022a, 2022b). For example, developmental instability correlates with phenotypic plasticity in wing size and shape of *Drosophila simulans* in response to abiotic environmental (nutrient, temperature, and light) stresses (Saito, Tsuboi, and Takahashi 2024). Developmental noise or instability helps the production of imperfect plasticity that benefits competition, which provides an evolutionary path that allows subsequent refining plasticity toward its optimum (Draghi 2020). Developmental instability can increase to facilitate adaptive plasticity before or during exposure to stress, leading to more positive correlations between developmental instability and plasticity (Saito, Tsuboi, and Takahashi 2024); or it can also decrease to stabilize performance after exposure to stress, when negative correlations between them increase, leading to non‐significant or negative relationships (Wang and Zhou 2022b). It is also possible that FA in leaf size will have stronger correlations with PI in leaf size, which was not measured in this study.

#### 4.2.2 Correlation Between Inter‐Individual Variation and Plasticity

Our results showed that CV_inter_ and PI of total mass were positively correlated in the early heterogeneous treatment (in the 1st round) but were negatively correlated after heterogeneous experience (in the 2nd round). This suggests that canalization can be advantageous or disadvantageous, depending on the circumstances, leading to complex relationships between canalization and plasticity, involving both positive and negative correlations (Wang and Zhou 2021, 2022b). Increased inter‐individual variability may facilitate positive plastic responses in more favorable conditions (Wang and Zhou 2021) or under moderate stresses (unpublished data). Under severe environmental stress, however, increased inter‐individual variation in traits may simply reflect being damaged, thus less positively correlated with plasticity (Wang and Zhou 2022b). In our study, early heterogeneous experience may not have been more stressful than later inundation or drought, thereby it is more probable that correlations between canalization and plasticity more likely to depend on whether they occurred before or after plastic response.

Our results suggested that the correlations between inter‐individual variation and plasticity depend on the induction of plasticity: positive correlations increase during early heterogeneous experiences, or before plastic responses; negative correlations increase after plastic responses. Plasticity and canalization (or developmental variability, robustness) both function as mechanisms that buffer against environmental variation and disturbance (Debat and David 2001; Meiklejohn and Hartl 2002; Waddington 1952, 1956). They may cooperate with or promote each other or reflect negative environmental effects. Plasticity can also drive developmental variation through direct positive responses, or reflect the result of such variation within developmental systems (Parsons et al. 2020). A population with greater inter‐individual variation will be more able to produce adaptive (more positive or less negative) plasticity in traits in face of future environmental changes (Gibson and Dworkin 2004); thus, greater inter‐individual variation and plasticity are favored by selection in this situation (Walter et al. 2024), leading to increased positive correlations between them.

#### 4.2.3 Correlations Between Fluctuating Asymmetry and Inter‐Individual Variation

Our results showed a few positive correlations between FA and CV_inter_, which occurred for species experiencing an early heterogeneous experience or in late drought (Tables 3 and 4), which is consistent with other studies (Lazić et al. 2016; Willmore, Klingenberg, and Hallgrimsson 2005). This suggests that environmental stress decreases FA and CV_inter_ but can strengthen the correlation between them. This may be because the two mechanisms operated in concert in response to environmental changes. Others have found no correlation between within‐individual variation in seed size and floral developmental instability (Pélabon et al. 2021). However, individuals of *Triatoma infestans* exposed to a sublethal dose of deltamethrin have larger, less symmetric, and less canalized wings (Nattero et al. 2021), implying positive correlation between developmental stability and canalization. Considered together, these studies suggest a great deal of complexity in the relationship between mechanisms of developmental stability and canalization. Regardless, developmental stability and canalization both emerge as by‐products of regulatory complexity and redundancy in developmental systems (Siegal and Bergman 2002), and thus may share some overlapping developmental bases in response to environmental variation.

#### 4.2.4 Correlations Between Intra‐Individual and Inter‐Individual Variation

Although intra‐individual variation is also often used as an indicator of developmental stability or variability, there was no correlation between leaf CV_intra_ and FA. We found one negative correlation between CV_intra‐LS_ and CV_inter‐TM_ initially, and one positive correlation between these variables later in drought condition (Tables 3 and 4). The negative correlation between CV_intra_ and CV_inter_ in leaf size has been reported for plants across a range of population densities and growth stages (Wang and Zhou 2022b). This suggests some association between CV_intra_ and FA, despite reflecting developmental stability in different ways, and that the complexity of the relationship between CV_intra_ and CV_inter_, can be affected by other factors.

## 5 Conclusion

Our results showed a few positive correlations between FA and CV_inter_ for species with early heterogeneous experience or in late drought, no correlations between FA, CV_intra_ and PI, and mixed results for correlations between CV_intra_ and CV_inter_ and between CV_inter_ and PI. Together, our results suggest that these relationships are important, but very complex and can be affected by many factors. Greater inter‐individual variability will more likely promote plastic responses before plasticity is induced, leading to increased positive (or less negative) correlations between them, whereas higher canalization may reflect phenotypic convergence due to produced plastic responses, and thus more negative correlations. Both higher FA and CV_intra_ can indicate faster growth or in better environments, but CV_intra_ may also simply reflect plant growth stage. Environmental stress decreased FA and CV_inter_, but strengthened correlations between them, since the two mechanisms might be more likely to cooperate under stress.

Our results provide direct evidence for relationships between fluctuating asymmetry, intra‐individual variation (developmental stability), inter‐individual variation (canalization) and plasticity in plant species, but also showed the complexity of their relationships. Increasing the number of traits studied should provide more clarity for these relationships. Future studies conducted with more plant species are also needed to explore different potential mechanisms and their relationships under different contexts of environmental heterogeneity at multiple growth stages. The complexity of these relationships suggests plants can deal with environmental changes in integrative ways which can be affected by many factors. Plants may evolve not simply in one or two directions but can self‐adjust ceaselessly while tracing environmental signals that vary throughout their lifetimes.

## Author Contributions

**Shu Wang:** conceptualization (lead), data curation (lead), formal analysis (lead), funding acquisition (equal), investigation (lead), methodology (lead), project administration (lead), resources (lead), software (lead), supervision (lead), validation (lead), visualization (lead), writing – original draft (lead), writing – review and editing (equal). **Ragan M. Callaway:** conceptualization (supporting), funding acquisition (equal), investigation (supporting), methodology (supporting), project administration (supporting), resources (supporting), software (supporting), writing – review and editing (equal).

## Conflicts of Interest

The authors declare no conflicts of interest.

## Supporting information


Data S1.

## Data Availability

Data are available from Dryad Digital Repository at: https://doi.org/10.5061/dryad.nzs7h44v6.
