# Supplementary material for: Associations Between Developmental Stability, Canalization, and Phenotypic Plasticity in Response to Heterogeneous Experience
Source: Ecol Evol. 2024 Oct 22;14(10):e70436. doi: 10.1002/ece3.70436 (PMC11494154; doi:10.1002/ece3.70436)
Supplement: Supplementary file 1 — Data S1. [file ECE3-14-e70436-s001.pdf]

Supporting Information

Table S1 Formulas for FA indexes (Palmer and Strobeck 1986, 1994, 2003) used in this study. *R* and *L* were the widths of right and left sides of a leaf, *n* was the total number of leaves, and *LS* (leaf size) was calculated by (*R*+*L*)/*2*. MS_{sj} was the mean squares of side × individual interaction, MS_m was the mean squares of measurement error, *M* was the number of replicate measurements per side, from a side × individual ANOVA on untransformed replicate measurements of *R* and *L*.

Index	Formula
FA_1	$mean\,\lvert R - L \rvert$
FA_2	$mean\,(\lvert R - L \rvert / LS)$
FA_3	$mean\,\lvert R - L \rvert / mean\,LS$
FA_4	$0.798 \times \sqrt{}\mathrm{var}\,(R - L)$
FA_5	$0.798 \times [\sum(R - L)^2 / n]$
FA_6	$0.798 \times \sqrt{}\mathrm{var}\,[(R - L) / LS]$
FA_7	$0.798 \times \sqrt{}\mathrm{var}\,(R - L) / mean\,LS$
FA_8	$mean\,\lvert \ln(R/L) \rvert$
FA_{10}	$0.798 \times \sqrt{}\,(MS_{sj} - MS_m) / M$

Table S2 Descriptive statistics of leaf size ([*R* + *L*] / 2) and FA for eight species after early heterogeneous and homogeneous experiences. N is the number of individual values; significant values for the slope from regression of |R - L| versus leaf size were marked by *. MS_m = measurement error mean square, σ_i^2 = non-directional asymmetry, df = approximate degree of freedom for non-directional asymmetry after partitioning out measurement error. The abbreviations for all species are in Table 2.

EX	SP	N	(R+L)/2	|R-L| vs. LS	R - L			|R-L|=FA_1	FA_{10}		
			Mean±SE	Slope±SE	Mean±SE	Skew±SE	Kurtosis±SE	Mean±SE	MS_m	σ_i^2	df
E_{hom}	C	49	14.23±0.61	0.005±0.02	0.082±0.10	-0.074±0.34	-1.005±0.67	0.61±0.05	0.003	0.21	2.24
	Lv	48	14.71±0.82	0.07±0.02*	0.065±0.15	-0.218±0.34	-0.218±0.67	0.82±0.09	0.003	1.73	2.00
	Pr	48	13.50±0.69	0.07±0.03*	-0.093±0.12	0.032±0.64	2.002±1.18	0.69±0.10	0.001	0.21	2.22
	Lc	124	40.09±9.26	0.008±0.01	0.027±0.18	0.636±0.22	1.193±0.43	1.53±0.11	1.05	2.39	1.76
	H	102	41.76±8.28	0.02±0.02	0.073±0.21	-0.919±0.23	5.582±0.46	1.63±0.14	0.57	1.20	2.24
	G	82	32.48±7.26	0.03±0.02	0.506±0.22*	0.977±0.27	2.473±0.53*	1.54±0.17	0.82	1.37	3.06
	Pa	88	35.68±3.54	0.05±0.02*	-1.543±0.41*	0.222±0.26	-0.752±0.51*	3.45±0.24	0.054	15.45	3.25
	A	81	37.80±3.54	0.13±0.03*	-0.512±0.48	0.054±0.37	-0.575±0.53	3.54±0.28	0.02	30.88	2.75
E_{het}	C	74	10.63±0.37	0.037±0.01*	-0.039±0.08	-0.514±0.28	1.88±0.55	0.49±0.05	0.008	0.13	3.29
	Lv	81	11.32±0.41	0.039±0.01*	0.216±0.08*	-0.32±0.27	1.486±0.53*	0.58±0.06	0.003	0.20	3.69
	Pr	81	9.14±0.36	0.045±0.01*	0.050±0.07	0.081±0.27	-0.309±0.53	0.50±0.04	0.110	0.05	0.80
	Lc	170	58.68±2.00	0.043±0.02*	-0.058±0.30	-0.345±0.19	0.729±0.37	3.02±0.19	2.02	3.09	2.34
	H	148	49.12±1.48	0.045±0.01*	0.298±0.22	0.210±0.20	0.488±0.40	2.14±0.14	0.65	2.56	3.32
	G	138	44.03±1.99	0.009±0.01	0.163±0.22	0.350±0.21	3.699±0.41	1.84±0.16	0.25	3.98	3.99
	Pa	202	67.41±2.26	0.028±0.02	0.374±0.36	0.126±0.17	-0.296±0.34	4.03±0.22	0.54	40.56	4.19
	A	189	56.09±1.78	0.011±0.01	0.756±0.33*	0.059±0.18	0.138±0.35*	3.59±0.21	0.001	25.71	4.25

Table S3 The results (mean squares [*MS*] and *F*-values) of two-way ANOVA for all between-sides variation relative to measurement error, leaf side (S), leaf (L), individual (I), measurement (M) and side and individual interaction (S × I) on the width of leaves for each species after early heterogeneous and homogeneous experiences. Abbreviations for all traits are in Table 2.

SP	Sources of variation	E_{hom}				E_{het}			
		Df	*MS*	*F*	*p*	Df	*MS*	*F*	*p*
C	S	1	1.656	4.822	0.030	1	3.906	5.408	0.021
	I	17	9.030	26.295	<0.001	19	17.023	23.568	<0.001
	L	4	3.298	9.602	<0.001	4	1.752	2.425	0.050
	S × I	17	0.621	1.809	0.032	19	1.302	1.802	0.025
	L × I	4	0.846	2.463	0.048	4	0.732	1.014	0.402
	M	1	0.016	0.045	0.832	1	0.172	0.238	0.627
Lv	S	1	32.119	12.325	0.001	1	16.894	6.528	0.011
	I	19	61.940	23.767	<0.001	19	51.985	20.089	<0.001
	L	4	27.392	10.511	<0.001	4	56.306	21.759	<0.001
	S × I	19	4.773	1.832	0.019	19	3.660	1.414	0.143
	L × I	4	1.040	0.399	0.809	4	4.707	1.819	0.127
	M	1	0.002	0.001	0.975	1	0.005	0.002	0.964
Pr	S	1	3.914	9.914	0.002	1	9.236	19.812	<0.001
	I	19	13.444	34.054	<0.001	19	4.635	9.943	<0.001
	L	4	6.898	17.472	<0.001	4	0.276	0.593	0.668
	S × I	19	0.504	1.275	0.199	19	0.476	1.021	0.436
	L × I	4	0.127	0.321	0.864	4	0.137	0.295	0.881
	M	1	0.018	0.046	0.830	1	0.161	0.345	0.557
Lc	S	1	0.995	0.278	0.598	1	0.913	0.436	0.510
	I	18	108.073	30.202	<0.001	18	115.543	55.137	<0.001
	L	4	20.627	5.764	<0.001	4	6.324	3.018	0.018
	S × I	18	3.869	1.081	0.371	18	1.071	0.511	0.952
	L × I	4	5.943	1.661	0.159	4	1.282	0.612	0.655
	M	1	0.690	0.193	0.661	1	0.002	0.001	0.972
H	S	1	0.145	1.291	0.257	1	1.814	17.819	<0.001
	I	16	7.122	63.606	<0.001	16	10.025	98.471	<0.001
	L	4	0.968	8.646	<0.001	4	0.178	1.753	0.139
	S × I	16	0.103	0.923	0.543	16	0.163	1.597	0.069
	L × I	4	0.044	0.393	0.813	4	0.256	2.512	0.042
	M	1	0.042	0.377	0.540	1	0.130	1.281	0.259
G	S	1	16.148	8.461	0.004	1	2.154	1.884	0.171
	I	19	67.051	35.132	<0.001	18	32.536	28.454	<0.001
	L	4	14.060	7.367	<0.001	4	14.221	12.437	<0.001

	S × I	19	3.966	2.078	0.006	18	3.532	3.089	<0.001
	L × I	4	0.614	0.322	0.863	4	0.062	0.054	0.995
	M	1	0.295	0.155	0.694	1	0.392	0.342	0.559
Pa	S	1	6.064	4.062	0.045	1	4.776	9.288	0.002
	I	18	12.687	8.500	<0.001	19	11.851	23.048	<0.001
	L	4	27.742	18.586	<0.001	4	4.860	9.451	<0.001
	S × I	18	0.714	0.478	0.965	19	0.278	0.540	0.944
	L × I	4	0.280	0.188	0.945	4	0.433	0.841	0.500
	M	1	0.021	0.014	0.905	1	0.022	0.043	0.836
A	S	1	0.453	0.515	0.474	1	0.054	0.130	0.718
	I	17	54.062	61.392	<0.001	19	33.032	79.040	<0.001
	L	4	9.864	11.202	<0.001	4	11.301	27.041	<0.001
	S × I	17	0.360	0.409	0.982	19	0.564	1.350	0.151
	L × I	4	0.348	0.395	0.812	4	0.820	1.962	0.100
	M	1	0.010	0.012	0.914	1	0.058	0.138	0.710

Table S4 *F*-values for three-way ANCOVA for the effects of early experience (EX), nativity (NA) and their interactions on mean values of leaf size, fluctuating asymmetry (FA_1, FA_2 and FA_{10}) and intra- and inter-individual coefficient of variations in leaf size ($CV_{intra-LS}$ and $CV_{inter-LS}$), for all species after the 1st round of treatments, with initial size (IS) as a covariate. Abbreviations for all traits are in Table 2. * $p < 0.05$, ** $p < 0.01$, *** $p < 0.001$

Source	df	LS	FA_1	FA_2	FA_{10}	$CV_{intra-LS}$	$CV_{inter-LS}$
IS	1	13.06***	2.43	29.63***	12.54***	31.38***	4.88*
EX	1	**4.12***	1.61	0.52	0.20	0.00	0.14
NA	1	**53.44*****	**13.49*****	**4.68***	0.01	0.58	2.14
EX × NA	1	0.03	0.01	0.00	0.03	0.44	0.12

Table S5 *F* values for two-way ANCOVA for the effects of early experience (EX), nativity (NA) and their interactions for CV_{inter} in shoot mass ($CV_{inter\text{-}SM}$), root mass ($CV_{inter\text{-}RM}$), total mass ($CV_{inter\text{-}TM}$) and root to shoot ratio ($CV_{inter\text{-}R/S}$) of all species after the 1st round of treatments. Values also for three-way ANCOVA on effects of early experience (EX), late conditions (LC) and nativity (NA) and their interactions on these variables after the 2nd round of treatments, with CV_{inter} of initial size ($CV_{inter\text{-}IS}$) as a covariate. * $p < 0.05$, ** $p < 0.01$, *** $p < 0.001$

Source	df	$CV_{inter\text{-}SM}$	$CV_{inter\text{-}RM}$	$CV_{inter\text{-}TM}$	$CV_{inter\text{-}R/S}$
After the 1st round					
$CV_{inter\text{-}IS}$	1	8.34*	7.27*	8.19*	1.70
EX	1	1.05	1.78	1.33	2.66
NA	1	**7.369***	0.80	2.34	0.33
EX × NA	1	0.13	0.36	0.18	0.31
After the 2nd round					
$CV_{inter\text{-}IS}$	1	0.54	1.95	1.07	3.60
EX	1	0.05	**5.02***	0.55	0.75
LC	3	2.71	2.89	2.74	0.68
NA	1	0.83	0.59	0.08	**4.13***
EX × LC	3	0.12	0.31	0.03	0.29
EX × NA	1	0.90	0.20	0.65	1.12
LC × NA	3	1.89	2.00	2.28	0.54
EX × LC × NA	2	0.77	0.77	0.96	0.20

Table S6 *F* values for four-way ANOVA for effects of early experience (EX), plasticity type (PT, plasticity in response to inundation or drought vs. moderate conditions), nativity (NA) and their interactions for relative plasticity in shoot mass (PI_{SM}), root mass (PI_{RM}), total mass (PI_{TM}), and root: shoot ($PI_{R/S}$) after the 2nd round of treatments. * $p < 0.05$, ** $p < 0.01$, *** $p < 0.001$

Source	df	PI_{SM}	PI_{RM}	PI_{TM}	$PI_{R/S}$
EX	1	0.02	0.02	0.06	0.20
PT	2	1.25	**16.28*** **	**9.42** **	**12.83** **
NA	1	1.01	1.43	1.40	0.05
EX × PT	2	0.20	0.64	0.61	0.15
EX × NA	1	0.16	0.28	0.33	0.60
PT × NA	2	1.23	1.99	1.38	0.39

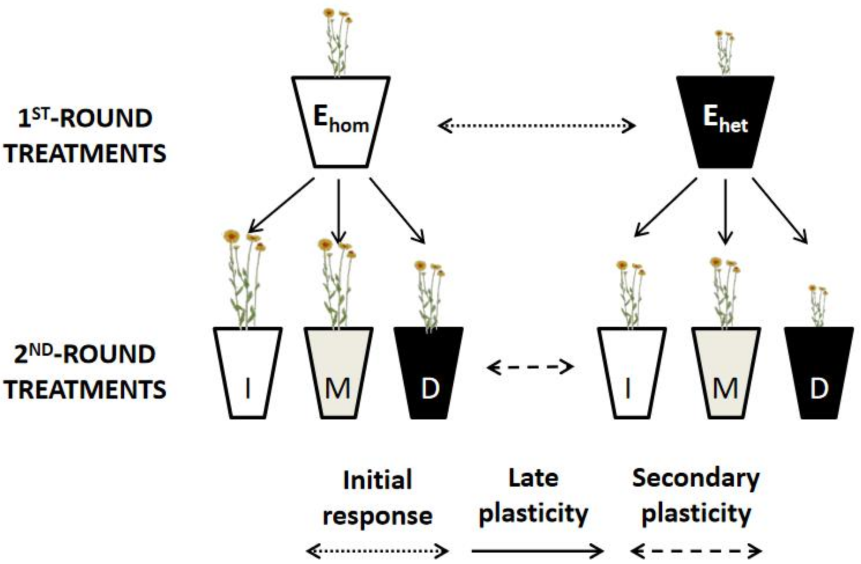

Fig. S1 Experimental design showing the 1st round of heterogeneous (E_{het}) and homogeneous (control, E_{hom}) water treatments, and the 2nd round of inundation (I), moderate (M) and drought (D) treatments used in this study. A subset of plants was harvested and measured after the 1st round of treatments, with all the other plants entering the 2nd round (Wang and Callaway 2022).

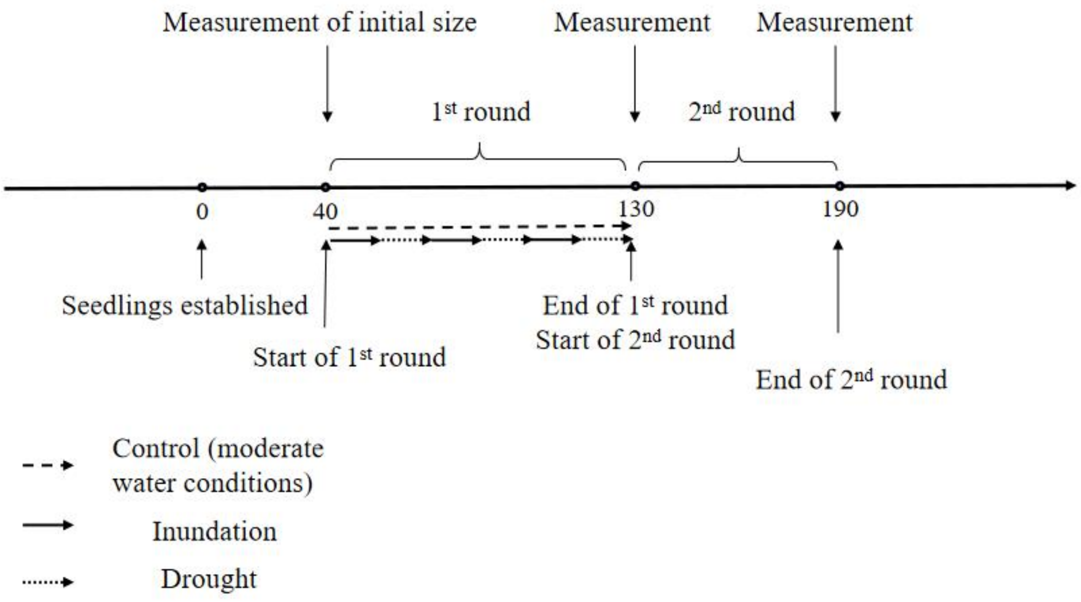

Fig. S2 The timing of the two rounds of treatments (Wang and Callaway 2022).

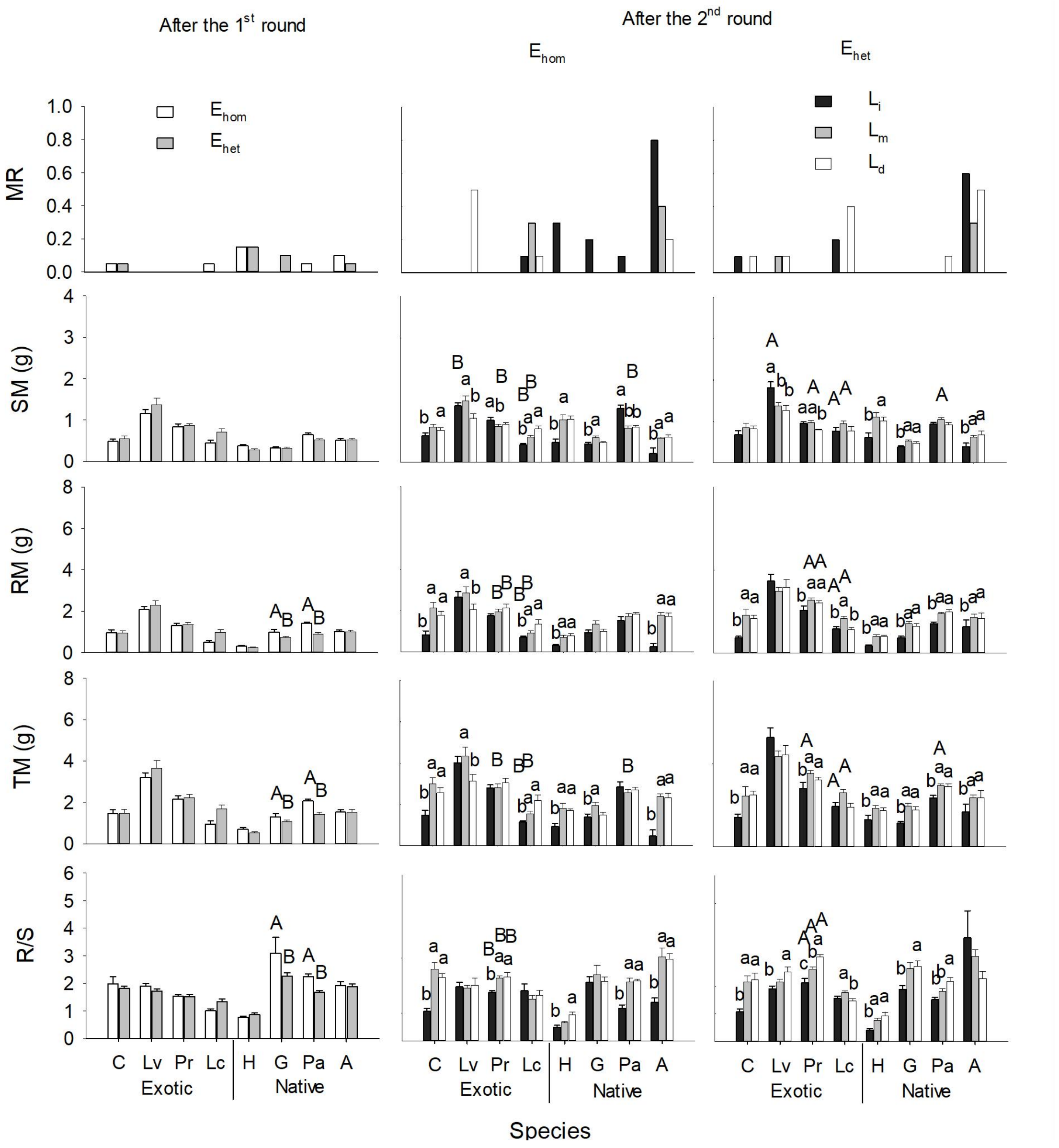

Fig. S3 Mortality rates (MR) and mean values (±SE) of shoot mass (SM), root mass (RM), total mass (TM) and root/shoot ratios (R/S) for eight species (grouped by exotics vs. natives) after the 1st round of heterogeneous (E_{het}) and homogeneous (E_{hom}) treatments, and performances of these traits in the 2nd-round inundation, moderate and drought conditions after the 1st-round treatments (Wang and Callaway 2022). Different lower-case letters denote differences due to the late treatments; different upper-case letters denote differences due to the early treatments ($p < 0.05$). The abbreviations for all species are in Table S1.

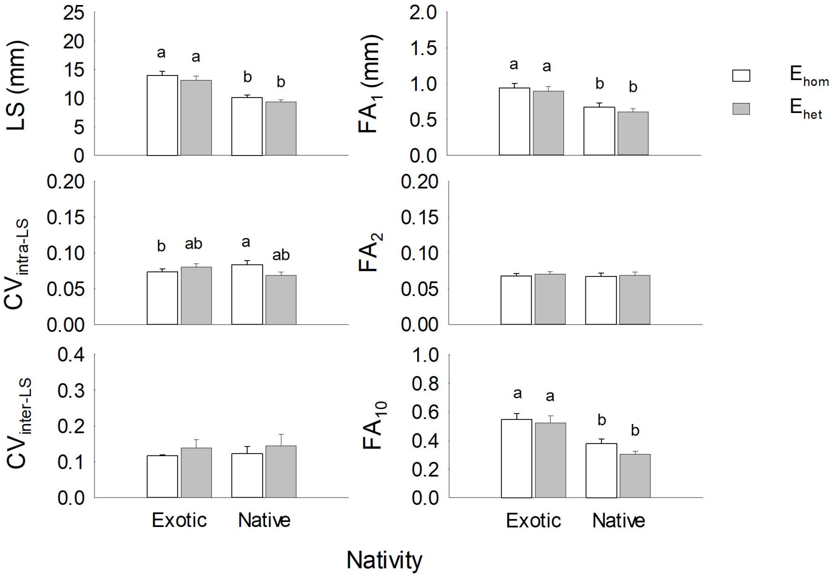

Fig. S4 Mean values (±SE) of leaf size (LS), indexes of leaf fluctuating asymmetry (FA_1, FA_2, FA_{10}), intra- and inter-individual coefficient variation in leaf size ($CV_{intra-LS}$ and $C_{inter-LS}$) in LS for exotic and native species in the 1st round of heterogeneous (E_{het}) and homogeneous (E_{hom}) treatments. Different letters indicate significant differences between E_{het} and E_{hom} treatments for each species ($p < 0.05$).

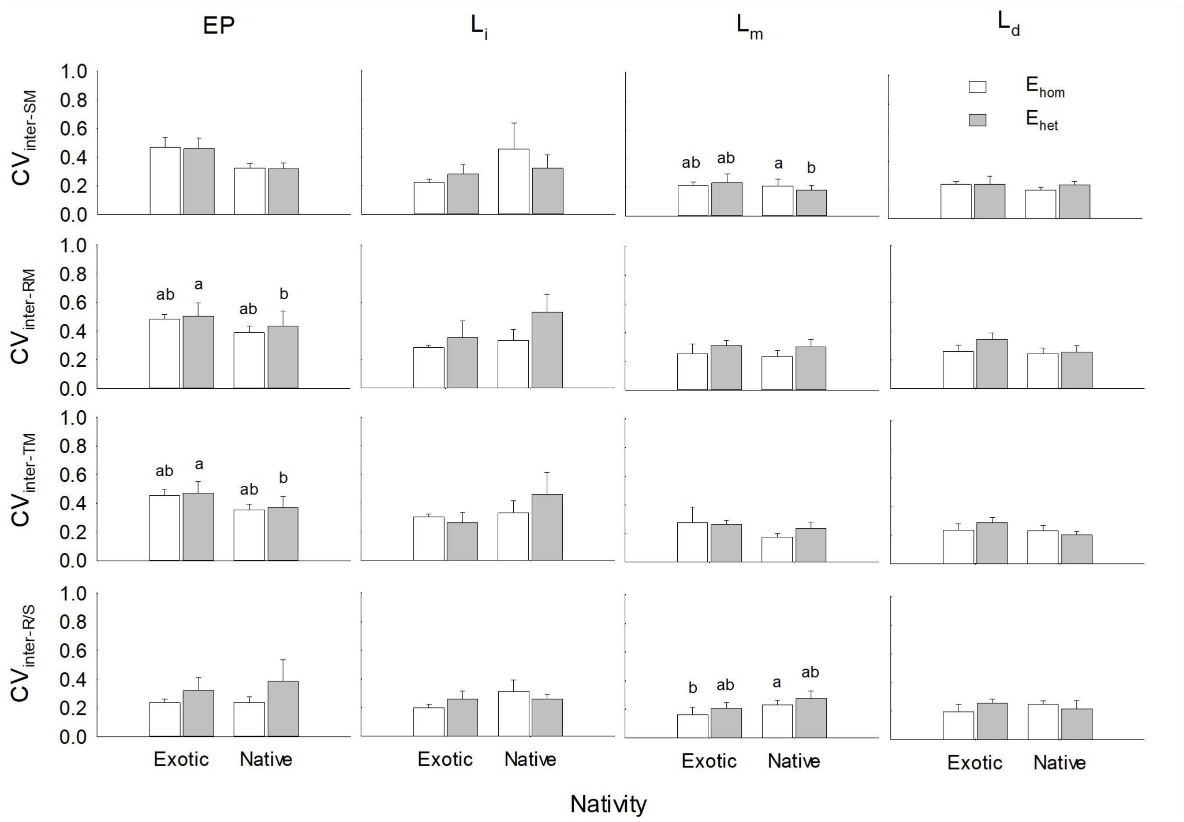

Fig. S5 Mean values (± SE) of inter-individual coefficient variation (CV_{inter}) in shoot mass ($CV_{inter-SM}$), root mass ($CV_{inter-RM}$), total mass ($CV_{inter-TM}$) and root to shoot ratio ($CV_{inter-R/S}$) in the 1st round of homogeneous (E_{hom}) and heterogeneous (E_{het}) treatments (EP), and in the 2nd round of inundation, moderate water and drought conditions (L_i, L_m and L_d) for exotic and native species with early E_{hom} and E_{het} experiences. Different letters indicate significant differences between E_{het} and E_{hom} treatments or between exotic and native species ($p < 0.05$).

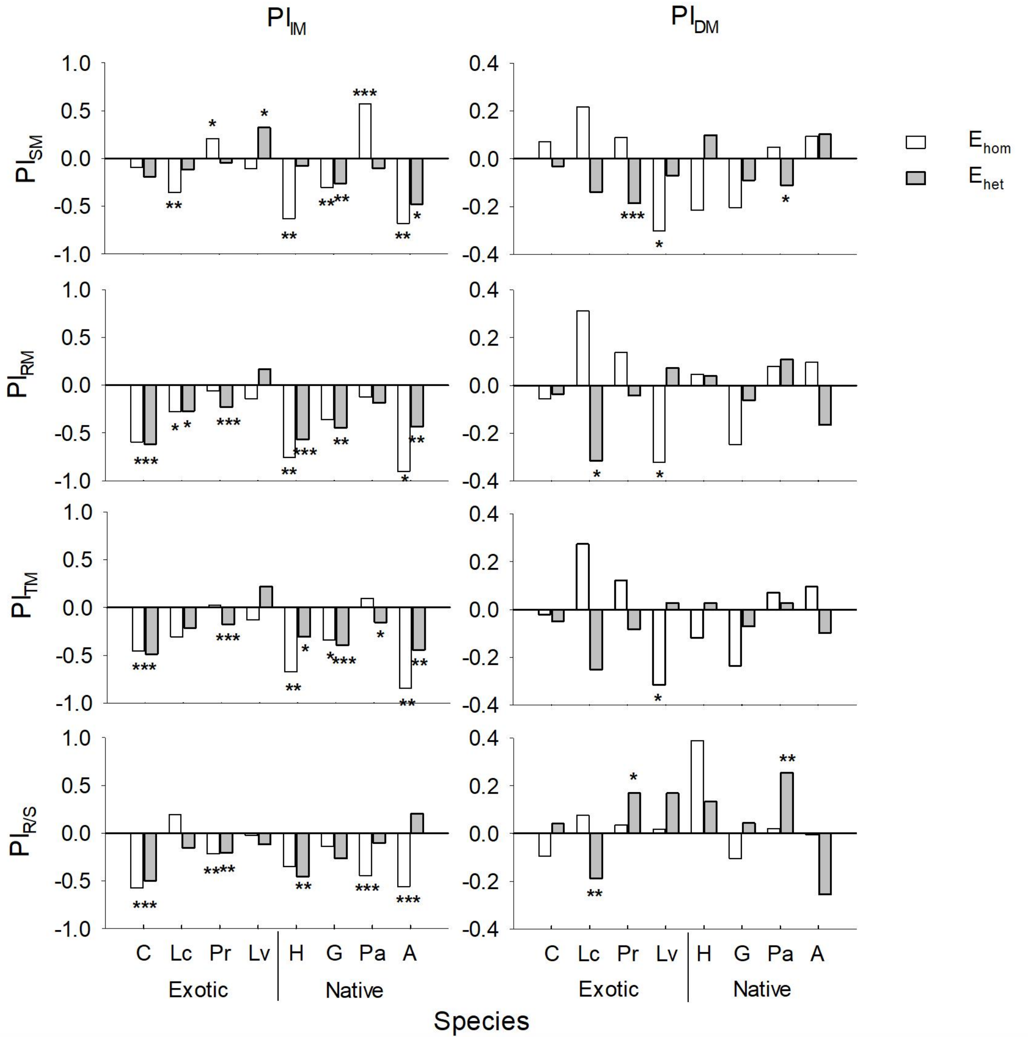

Fig. S6 Relative plasticity (PI_{rel}) in shoot mass (PI_{SM}), root mass (PI_{RM}), total mass (PI_{TM}) and root to shoot ratio ($PI_{R/S}$) in response to inundation or drought vs. moderate conditions (PI_{IM} and PI_{DM}) for eight species (grouped by exotics vs. natives) after the 1st-round treatments of heterogeneous (E_{het}) and homogeneous (E_{hom}) experience. Significance levels (* $p < 0.05$, ** $p < 0.01$, *** $p < 0.001$) were marked for PI values the ANCOVAs for effects of the 2nd round of treatments on mean trait values for each species (Fig. S3). The abbreviations for all species are in Table 2.
